# A Comparative Study of Dairy and Non-Dairy Milk Types: Development and Characterization of Customized Plant-Based Milk Options

**DOI:** 10.3390/foods13142169

**Published:** 2024-07-09

**Authors:** Aline Rolim Alves da Silva, Ricardo Erthal Santelli, Bernardo Ferreira Braz, Marselle Marmo Nascimento Silva, Lauro Melo, Ailton Cesar Lemes, Bernardo Dias Ribeiro

**Affiliations:** 1 Instituto de Química, Universidade Federal do Rio de Janeiro, Av. Athos da Silveira Ramos, 149, Bloco A—Cidade Universitária, Rio de Janeiro 21044-020, RJ, Brazil; alinerolimas@gmail.com (A.R.A.d.S.); santelli@iq.ufrj.br (R.E.S.); bernardobraz@pos.iq.ufrj.br (B.F.B.); marsellemarmo@hotmail.com (M.M.N.S.); 2 Escola de Química, Universidade Federal do Rio de Janeiro. Av. Athos da Silveira Ramos, 149, Bloco E—Cidade Universitária, Rio de Janeiro 21044-020, RJ, Brazil; lauro@eq.ufrj.br (L.M.); ailtonlemes@eq.ufrj.br (A.C.L.)

**Keywords:** water-soluble extract, non-milk products, mixture planning, technological properties, shelf life

## Abstract

Plant-based milk has gained considerable attention; however, its high nutritional variation highlights the need for improved formulation designs to enhance its quality. This study aimed to nutritionally compare cow milk with plant-based milk produced from hazelnuts (H), Brazil nuts (BN), cashew nuts (CN), soybeans (S), and sunflower seeds (SS), and to perform physicochemical and technological characterization. The plant-based milk produced with isolated grains showed a nutritional composition inferior to that of cow milk in almost all evaluated parameters, protein content (up to 1.1 g 100 g^−1^), lipids (up to 2.7 g 100 g^−1^), color parameters, minerals, and especially calcium (up to 62.4 mg L^−1^), which were originally high in cow milk (up to 1030 mg L^−1^). However, the plant-based milk designed using a blend composition was able to promote nutritional enhancement in terms of minerals, especially iron (Fe) and magnesium (Mg), high-quality lipids (up to 3.6 g 100 g^−1^), and carbohydrates (3.4 g 100 g^−1^ using CN, BN, and S). The protein content was 1.3% compared to 5.7 in cow milk, and the caloric value of plant-based milk remained 32.8 at 52.1 kcal, similar to cow milk. Satisfactory aspects were observed regarding the shelf life, especially related to microbiological stability during the 11 d of storage at 4 °C. For the designed plant-based milk to be equivalent to cow milk, further exploration for optimizing the blends used to achieve better combinations is required. Furthermore, analyzing possible fortification and preservation methods to increase shelf life and meet the nutritional and sensory needs of the public would be interesting.

## 1. Introduction

The increase in the public’s awareness of health has led to the search for and increased consumption of foods that not only nourish but also offer physiological benefits to the human body, including the potential prevention of cancers, cardiovascular diseases, and neurodegenerative conditions, and support for healthy aging [1]. In this regard, plant-based foods and beverages have been constantly discussed owing to their nutritional properties as well as aspects that encourage the substitution of animal-derived products, whether for health reasons, animal welfare, environmental concerns, religious beliefs, or lifestyle changes [2].

The global market for plant-based foods has been expanding substantially, prompting the industry to develop ingredients and products that meet the sensory, technological, and, most importantly, nutritional needs of consumers, as well as the sector that uses them as ingredients in food processing. The plant-based market is expected to grow to USD 160 billion by 2030, compared to the current USD 30 billion in 2023, indicating a considerable need for the development of ingredients, foods, and beverages [3].

Among plant-based products, plant-based milk stands out as a potential alternative for people seeking non-dairy products, especially for those intolerant to lactose and/or allergic to cow milk proteins, in addition to the choices and restrictions mentioned earlier. Furthermore, these products generally have lower environmental footprints, require less water, and emit fewer greenhouse gases during their production. Additionally, they often utilize land more efficiently, reducing deforestation pressures compared with dairy farming [4,5].

Plant-based milk comprises colloidal suspensions or emulsions of vegetable materials dissolved and disintegrated in water. They are prepared by disintegrating the vegetable raw material into a paste by mechanical force to remove coarse particles and subsequent homogenization [6,7]. Additionally, it is necessary to incorporate additives, stabilization, and adequate storage to improve shelf life and food safety [8].

In general, plant-based milk is nutritionally deficient and is difficult to standardize compared to cow milk. Even products produced from the same plant can exhibit high levels of nutritional and sensory variability [9]. Plant-based sources are considered functional nutraceutical foods because they are rich in bioactive components (minerals, non-allergenic proteins, and essential fatty acids) [10]. Plant-based milk can be obtained from many different plants that contain substantial amounts of proteins, fibers, phenolic compounds, unsaturated fatty acids, and bioactive compounds such as phytosterols and isoflavones [8], and components with significant bioactive and technological properties [4]. Furthermore, plant-based milk does not contain specific components traditionally found in cow milk, such as cholesterol, saturated fatty acids, antigens, and lactose, which are not beneficial to everyone [11]. Globally, most plant-based milk is produced from soybeans, rice, oats, and almonds, with soy being the most popular raw material [12].

Among the aspects to be highlighted and overcome in non-milk products, especially plant-based milk, is the (i) need to offer products with sensory aspects similar to cow milk regarding color, texture, and, when possible, flavor [13]; (ii) many of the vegetables used in the production of plant-based products have undesirable off-flavors and antinutrients, requiring additional treatments, especially thermal treatments to reduce these aspects, which may impact their nutritional value [14,15]; (iii) negative perceptions have been created with the early introduction of low-quality plant-based products into the market, as was the case with plant-based milk [16]; and also (iv) the lack of uniform regulation on these products, which can compromise standardization and, primarily, product safety, creating risks for consumers and industries [17].

For plant-based milk to be considered a potential substitute for cow milk, it must have a similar nutritional composition that does not occur naturally. Therefore, improving its composition, which is usually achieved through fortification or formulation optimization using ingredient blends, is necessary. Some types of dual blends of plant-based milk have already been studied, such as rice and soy [18,19], soybean and tiger nuts [20], soybeans and Brazil nuts [21], Brazil nuts and macadamia [22], and soybeans and almonds [11]. More recently, ternary mixtures using a combination of melon seeds, peanuts, and coconuts were developed, which made it possible to identify flavors and evaluate the effects of ingredients on the sensory aspects of the product. An important aspect raised is the need to continuously design and optimize plant-based milk formulations with sustainable characteristics to offer products that are compatible and nutritionally adequate for the diet and food needs of the population [23]. However, no previous reports have been published on the production and characterization of water-soluble extracts from ternary mixtures using a combination of hazelnuts (H), Brazil nuts (BN), cashew nuts (CN), soybeans, and sunflower seeds (SS).

This study aimed to explore the feasibility of using unexplored plant-based ternary mixtures for the production of plant-based milk as an alternative to cow milk and to evaluate their physicochemical and technological properties and shelf life. More broadly, this study aimed to develop innovative and sustainable alternatives to cow milk in response to the increasing demand for plant-based products within the food industry.

## 2. Materials and Methods

**Figure 1:**
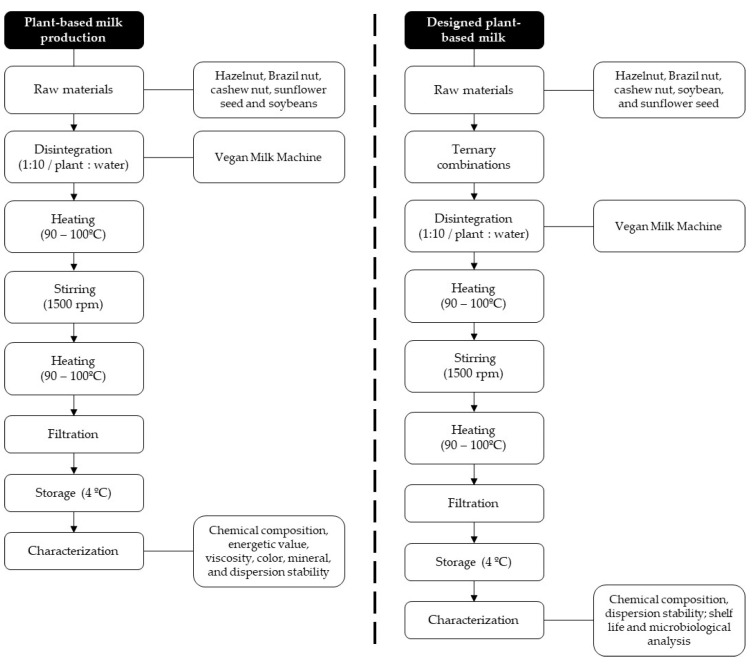
Generic flowchart for characterization of plant-based milk and designed plant-based milk (blends).

Plant-based milk and the designed plant-based milk (blend) were obtained according to the flowchart shown in Figure 1. This study focused on the production and characterization of plant-based milk produced from hazelnuts, Brazil nuts, cashews, soybeans, and sunflower seeds, and performed a comparative analysis with commercially available cow milk. For standardization, the terminology “plant-based milk” was adopted for the product obtained from the use of a single raw material separately, and the terminology “designed plant-based milk” was adopted for milk produced from the mixture of different raw materials to improve the physical, chemical, nutritional, and technological properties.

### 2.1. Material

The hazelnut (*Corylus avellana*), Brazil nut (*Bertholletia excelsa*), cashew nut (*Anacardium occidentale*), sunflower seed (*Helianthus annuus*), soybeans (*Glycine max*), cow milk (three brands of whole milk standardized at 3% fat and coded as N, EW, and QW, and three brands of skim milk with fat content below 0.5% and coded as M, ES, and QS), and mineral water were purchased at the local market.

### 2.2. Plant-Based Milk Alternative Production Using Isolated Raw Materials

Plant-based milk was prepared according to the methods of Zhou et al. [24] and Silva et al. [25] with modifications. The same process and conditions were used for each raw material separately (hazelnuts (H), cashew nuts (CN), Brazil nuts (BN), soy (S), and sunflower seeds (SS)). For this purpose, one gram of each raw material was weighed and placed in the Vegan Milk Machine (Polishop^®^) with 1000 mL of mineral water. The process consisted of 15 min of heating (90–100 °C), followed by 10 min of intercalated stirring at 1500 rpm (1 min each stirring interval), and then 2 min of heating. After the preparation was finished, the beverage was sieved and stored in a plastic bottle at 4 °C for further analysis.

### 2.3. Designed Plant-Based Milk

The designed plant-based milk was obtained from vegetable blends (H, BN, CN, S, and SS) in sufficient quantities to produce the same estimated composition as the cow milk used in the study. The same procedure described in Section 2.2 was adopted to obtain the designed plant-based milk. However, in this case, the ternary mixtures are described in Table 1.

### 2.4. Physicochemical Composition of Cow Milk, Plant-Based Milk, and Designed Plant-Based Milk

#### 2.4.1. Chemical Composition

The chemical composition was performed to determine the moisture, ash, and lipid content using the method described by the AOAC [26] and proteins using the Bradford [27] method. The carbohydrate content was calculated by subtracting lipid, protein, fiber, moisture and ash from 100%. The energetic value was calculated based on the composition using Atwater conversion factors of 4, 9, and 4 kcal/g for protein, lipids, and carbohydrates, respectively [28].

#### 2.4.2. Mineral Composition Determination

The mineral contents of calcium (Ca), iron (Fe), phosphorus (P), magnesium (Mg), potassium (K), and zinc (Zn) in the samples were determined using Inductively Coupled Plasma Optical Emission Spectrometry (ICP-OES). Quantification was performed via interpolation using an analytical curve with four standard solutions for calibration. Solutions of Ca, Fe, K, Mg, P, and Zn were obtained from the dilution of SpecSol stock standard solution at a concentration of 1000 or 10,000 mg L^−1^ (Quimlab Química & Metrologia^®^, Jacareí, São Paulo, Brazil) until obtaining the desired concentrations using matrix matching and ultrapure water obtained from a Milli-Q^®^ system, model Direct 8 (Merck Millipore, Burlington, MA, USA). The operational conditions for ICP-OES used in the determination of mineral nutrients in samples were as follows: incident power, 1200 W; plasma gas flow, 12 L min^−1^; coating gas flow, 0.2 L min^−1^; nebulization gas flow, 0.02 L min^−1^; nebulizer pressure, 1.0 bar; sample introduction flow rate, 1.0 mL min^−1^; integration time, 1 s; high resolution; and wavelength (nm): Ca = 396.847; Fe = 259.940; K = 766.490; Mg = 279.553; P = 214.914; Zn = 213.856.

### 2.5. Stability and Shelf Life Analysis

The stability and shelf life were determined through the assessment of various parameters during storage at 4 °C and 24 °C for 12 d. The analyses were performed on day 1, 6, and 12 of storage to measure pH and viscosity (viscometer digital Marte MVD-20), with a L1 measuring spindle at a constant shear rate of 50 rpm and range of 14–150 s^−1^ [29]. Color, based on the Commission Internationale de l’Eclairage (CIE) system, was expressed as L* (lightness or brightness), a* (redness/greenness), and b* (yellowness/blueness) [30]. The dispersion stability was determined by the percentage of separation at 4 °C and 25 °C for 12 d [31], considering the formation of a separation line between the bottom phase and the top phase. The percentage of separation was determined using Equation (1):

(1) $$Dispersion\;stability=\frac{\left(Total\;height\;of\;the\;plant\;based\;milk-Condensed\;dispersed\;phase\right)}{Total\;height\;of\;the\;plant\;based\;milk}\times 100$$

Microbiological characteristics were assessed using enumerating mesophilic and psychrotropic aerobic bacteria. Both analyses were performed using counting agar. Mesophilic bacteria were incubated at 35 °C for 2 d, and psychotropic bacteria were incubated at 4 °C for 10 d. The analysis was performed as described by Silva et al. [32], and the results were expressed as colony-forming units per mL (CFU/mL).

### 2.6. Statistical Analysis

One-way analysis of variance (ANOVA) was performed for the chemical and mineral analyses of the 12 samples, in addition to shelf life analysis. Statistical analysis was performed in triplicate, and the results are expressed as means. Tukey’s test (*p* ≤ 0.05) was used as a post hoc test to identify significant differences, when necessary. Principal component analysis (PCA) with Pearson’s correlation matrix and Agglomerative Hierarchical Cluster Analysis were performed based on the Euclidean distance using Ward’s method. All analyses were conducted using the XLSTAT software, version 2018.6 (Addinsoft, Paris, France).

## 3. Results and Discussion

### 3.1. Comparative Analysis of Cow Milk and Plant-Based Milk

Table 2 shows the physicochemical compositions of cow milk and plant-based milk. In general, and consistently, it is possible to observe the high moisture content (88.1–95.0%), a critical characteristic for safety and shelf life because it can promote microbial growth and the occurrence of chemical and enzymatic reactions, compromising the safety and nutritional, sensory, and technological properties [33].

Regarding proteins, a deficiency was verified in all plant-based milk (0.7 to 1.1 g 100 g^−1^) when compared to cow’s milk (2.7 to 6.2 g 100 g^−1^), which is naturally rich in high-quality proteins [34]. In general, plant-based milk lacks the complete composition of essential amino acids found in cow milk. This can lead to inadequate protein intake, which is particularly concerning for groups with higher protein needs such as growing children, teenagers, pregnant or lactating women, and physically active individuals [35,36].

Plant-based milk presented a higher lipid content (2.6 to 4.5 g 100 g^−1^) than animal-origin products, including whole cow milk (~3.3 g 100 g^−1^). Lipids from nuts are rich in unsaturated fatty acids, antioxidants, and essential nutrients, offering various health benefits such as promoting cardiovascular health, protecting against free radical damage, improving skin and hair health, and regulating blood sugar levels [37,38,39].

Regarding carbohydrates, equal or lower concentrations are observed in plant-based milk (0.4 to 3.7 g 100 g^−1^) compared to cow milk (1.8 to 4.2 g 100 g^−1^). In terms of caloric content, both cow- and plant-based milk presented similar values. Cow milk ranges from 30.0 to 60.8 kcal per 100 mL of the product (as expected, higher values are observed for non-skimmed milk, thus with higher lipid content and caloric value), while the plant-based milk ranges from 32.8 to 52.1 kcal, with higher caloric values associated with nuts with higher lipid content in their composition (Brazil nuts and cashews have a lipid content exceeding 40%) [40,41].

When comparing cow milk and plant-based milk, it is evident that the L* consistently surpasses that of cow milk. This higher L* is characteristic of cow milk, which typically exhibits a whitish and opalescent appearance. When comparing whole cow milk and skim milk, higher brightness was observed in the former because it has a higher concentration of large fat globules that make it opaque and, therefore, appear brighter. The color of a product, especially plant-based milk, can influence consumers’ purchase intention and create expectations regarding its flavor [42]. The diminished brightness observed in plant-based milk is attributed to the elevated levels of pigmented compounds, including carotenoids, flavonoids, and proanthocyanidins, which can intensify the color of the products. Additionally, factors such as ingredient concentration and manufacturing processes, including raw material roasting, can influence the brightness of plant-based milk [43,44,45]. Color variation can also be attributed to differences in the size and concentration of the particulate matter present, which can interfere with light scattering, as well as the types and levels of chromophores [46].

Regarding viscosity, a higher value was observed for cashew nuts (23.3 cp). According to Oh and Lee [47], the higher viscosity of cashew nut milk compared to other plant-based milk can be attributed to their higher protein content, saturated fatty acid content, and higher carbohydrate concentration, among other factors.

The variation in plant-based milk and cow milk concerning all evaluated parameters, when compared to various other studies in the literature, can be attributed to numerous factors, including process parameters, plant material-to-water ratio, type and concentration of plant materials, varieties, and combinations. This highlights the importance of complete standardization and blending planning to offer products similar to conventional products and meet consumer needs [21,29].

Using the dendrogram (Figure S1) for cluster analysis, distinct groups were formed with a dissimilarity of 18%: whole milk and cashew milk (Group 1), skimmed milk (Group 2), and plant-based milk (Group 3). Through truncation, cashew nut milk and whole cow milk constituted a single group, thereby presenting a high similarity between them. These data were important because, among all the plant-based milk analyzed, cashew nut was the first in a group with cow milk, showing some similar characteristics.

**Figure 2:**
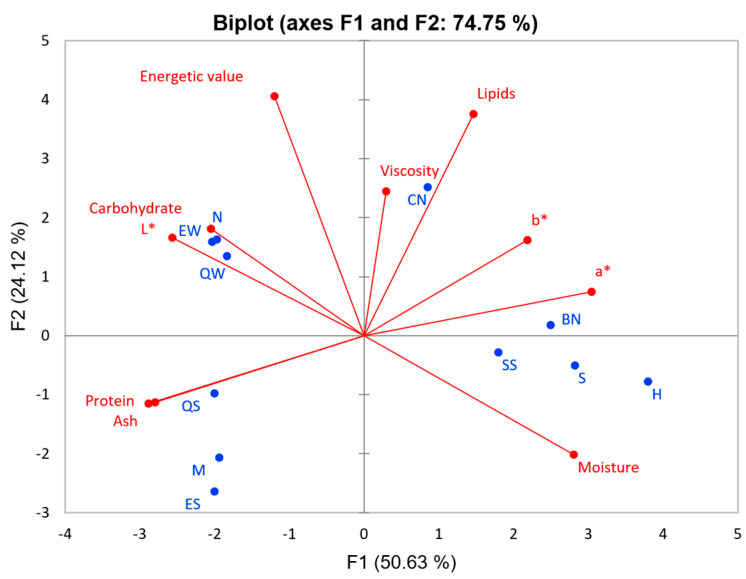
Principal components analysis of the composition and viscosity of plant-based milk substitute (hazelnut (H), Brazil nut (BN), cashew nut (CN), sunflower seed (SS), and soy (S)), whole milk (N, EW, and QW), and skimmed milk (M, ES, and QS). (PC1 × PC2). Active variables (●) and observations (●).

In the PCA (Figure 2), the first two principal components explained 74.75% of total variance. Cashew nut milk is characterized by lipid content and viscosity and is an intermediate between cow milk and other plant-based milk. According to the results obtained, it can be observed that the composition of cashew nut milk presented characteristics similar to cow’s milk.

Table 3 compares the average of the analyzed variables of the groups formed in the cluster analysis, where it is possible to observe the main characteristics of each group. Group 1 was characterized by high L* and low moisture values, whereas Group 2 had the lowest values of lipids, a* and b*. Group 3 had the highest a*, b*, and moisture content and the lowest L*, protein, and ash content. No significant differences in the viscosities of the three groups were observed.

### 3.2. Dispersion Stability

**Figure 3:**
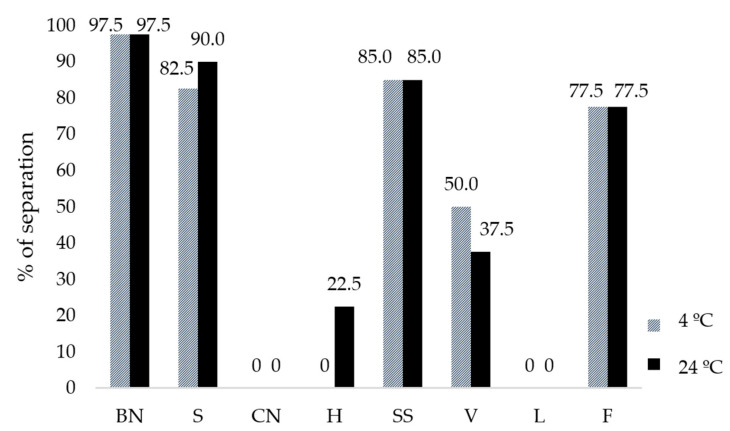
Phase separation index during 1 day of storage at 4 °C and 24 °C of plant-based milk (BN: Brazil nut; S: soy; CN: cashew nut; H: hazelnut; SS: sunflower seed) and designed plant-based milk (V: 69.57% CN + 17.29% S + 13.04% H; L: 85.71% CN + 9.52% SS + 4.76% BN; F: 57.14% CN + 38.10% BN + 4.76%S).

Figure 3 shows the phase separation index of the plant-based milk after 1 d of storage at 4 and 24 °C. Cashew nut milk during both storage temperatures exhibited good stability as there was no separation at any storage temperature (0% of separation), whereas hazelnut milk showed better stability at 4 °C than at 24 °C (0% and 22.5% separation, respectively). Soy, Brazil nut, and sunflower milk showed low stability since, at both storage temperatures, there was more than 80% separation.

In the case of hazelnut and soy-based milk, a higher percentage of separation is observed at a temperature of 24 °C. The storage temperature at 24 °C influenced the stability in some cases since low temperatures play an important role in maintaining the physical properties and stability of the emulsion. The stability of emulsions in plant-based milk is important for ensuring that the lipid and aqueous components remain uniformly dispersed over time without undesirable separation or coalescence.

Although some plant-based milks have higher protein compositions (such as soy and cashew nut), which are important components for binding and maintaining the association between water and oil, the absence of stabilizers appears to impair their ability to keep the dispersed phases evenly distributed [48,49,50].

In general, because no additives have been added and no process has been undertaken to increase shelf life, phase separation is expected. Emulsion breakdown is a phenomenon in which the dispersed phases of an emulsion separate over time, resulting in the formation of distinct layers or the coalescence of dispersed droplets. Several factors can lead to emulsion breakdown in plant-based milk, including physical instability such as temperature fluctuations, chemical instability, and sedimentation [48,51,52].

To prevent phase separation in plant-based milk, various options are available, including reducing the particle size through homogenization and adding stabilizers. Stabilizers in emulsions have potential benefits (stability, improved viscosity, and texture, among others) and can act in different ways, such as functioning as a physical barrier that prevents droplet coalescence, keeping them dispersed in the beverages, contributing to increased viscosity, which slows down the movement of fat droplets and their tendency to cluster and coalesce, steric hindrance through electrostatic repulsion between molecules, preventing their approach and separation, and interaction with the aqueous phase, contributing to the formation of a three-dimensional network that keeps the components evenly dispersed [53,54,55].

### 3.3. Mineral Composition

Adequate mineral intake is essential for maintaining the health and optimal functioning of the human body. Minerals such as Ca, Fe, K, Mg, P, and Zn play crucial roles in biological processes including the formation and maintenance of bones and teeth, the regulation of water and acid-base balance, nerve transmission, muscle contraction, and enzymatic function. Mineral deficiencies can lead to various health problems, such as osteoporosis, hypertension, and immune impairment, highlighting the need for a balanced diet rich in minerals to prevent disease and promote overall well-being [56]. Therefore, given the increasing demand for and consumption of this type of product, verifying the presence and composition of plant-based milk is essential. The mineral concentrations are presented in Table 4.

The most important mineral to be studied is Ca, as cow milk is the main source of Ca. Cow milk has a much higher concentration of Ca (up to 1030 mg L^−1^) than plant-based milk (up to 62.4 mg L^−1^). This indicates that plant-based milk is unable to supply dietary Ca and cannot be used as a substitute [57]. For this reason, many plant-based milk products are fortified with Ca to provide Ca-like amounts of cow milk because they are used as a substitute [58].

Regarding the Fe content, plant-based milk had higher concentrations than cow milk (up to 3.7 and <5.40 × 10^−3^ mg L^−1^, respectively). BN and CN are rich in Mg, thus their derivative milk is also rich in Mg (up to 141.0 and 202.0 mg L^−1^, respectively), being those with the highest concentration. Simultaneously, both cow milk (up to 84.4 mg L^−1^) and other plant-based milk have similar values (up to 81.9 mg L^−1^) [59].

Cow milk showed high values of K; whole milk B was the highest (2390.0 mg L^−1^), and SS milk showed values significantly close to some cow milk (862.0 mg L^−1^). However, other plant-based milks, in general, presented low values of potassium. Cow milk had the highest P (778.0 mg L^−1^), but CN milk was the only plant-based substitute that presented values significantly close to those of cow milk (342.0 mg L^−1^). BN and CN milk are rich in Zn (1.72 and 3.7 mg L^−1^, respectively), while both cow milk (except for whole milk A; 8.17 mg L^−1^) and other plant-based milks have similar values (up to 2.4 and 1.37 mg L^−1^, respectively).

Similar to the other parameters examined, a significant variation in the mineral composition was observed among the samples and in the data available in the literature. As previously described, this was expected because of differences in their compositions, as many plants present varieties and cultivars, or even the type of processing they undergo [6,60].

In addition to their nutritional implications, minerals play an important technological role in plant-based milk, as they can physically and chemically affect the stability of emulsions by influencing the ionic strength, electrostatic interactions of molecules, flocculation, and viscosity. Furthermore, minerals affect the flavor, acidity, and functionality of plant-based milk and can therefore be reintroduced into water to achieve the desired properties. Another option is supplementation using combinations of different plants to achieve an adequate composition of these components [46,61].

### 3.4. Comparison of the Physicochemical Composition of Cow Milk and Designed Plant-Based Milk

The designed plant-based milk was developed to achieve a nutritional composition similar to that of cow milk through a combination of different vegetables (Table 5). In general, cow milk has been found to have a higher ash content (up to 0.8 g/100 g^−1^ in skim milk C) compared to that of the plant-based milk (up to 0.34 g/100 g^−1^ in sample F which was composed of 57.14% CN, 38.10% BN, and 4.76% S). This has been previously reported and is related to the high concentrations of various minerals that have nutritional and technological impacts. Even for the developed plant-based milk, a much lower protein content was observed compared to cow milk (up to 1.3 and 6.2 g/L^−1^, respectively), which is naturally rich in high-quality proteins [34].

The components that most closely approximated the composition of cow milk were the lipid and carbohydrate contents because the vegetable composition was rich in these components. Except for skim milk, all the products had very similar lipid concentrations (~3.0 g/L). Additionally, the carbohydrate composition in the designed plant-based milk (2.7–3.4 g/L) remained within a range closer to that observed for cow milk (1.8–4.2 g/L). As previously reported, plant-based milk shows diminished brightness compared to cow milk. Similarly, skim cow milk appeared darker than whole milk, which was related to the higher brightness of cow milk owing to its whitish, opalescent appearance, with whole milk being brighter than skim milk because of its larger fat globule content.

**Figure 4:**
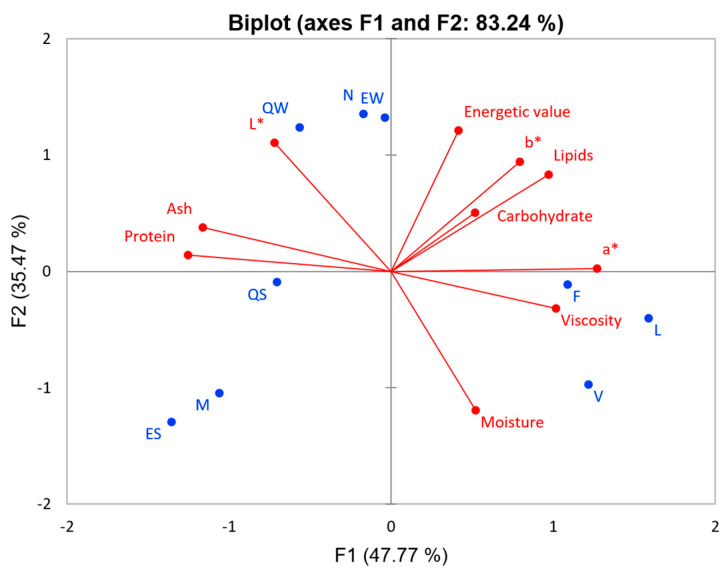
Principal components analysis of the composition and viscosity of designed plant-based milk, whole milk, and skimmed milk. (PC1 x PC2). Active variables (●) and observations (●).

The principal component analysis is shown in Figure 4. The first two principal components explained 83.24% of the total variance. At this point, it was possible to verify the three groups that formed (Figure S2): designed plant-based milk (Group 1), skim (Group 2), and whole cow milk (Group 3). A clear division between cow milk and plant-based milk was observed; therefore, their composition was different. None of the samples had a composition similar to cow milk. Figure S3 shows how the samples were gathered and indicates that the mixtures of plant-based milk and cow milk have different characteristics because they were placed in different groups.

Table 6 compares the averages of the analyzed variables of the groups formed in the cluster analysis. The primary characteristics of each group were observed. Group 1 was characterized by the highest moisture, viscosity, and a* content, and the lowest ash, protein, and L* content. Group 2 had the lowest lipid content, viscosity, and a* and b* values. Group 3 had the highest L* and lowest moisture contents. Ash, protein, and viscosity were not significantly different between Groups 2 and 3.

### 3.5. Comparison of the Physicochemical Composition of Plant-Based Milk and Designed Plant-Based Milk

The physicochemical compositions of the plant-based milk and the designed plant-based milk are presented in Table 5 (means within the same column with different uppercase letters between designed plant-based milk (L, V, and F) and plant-based milk (H, CN, BN, S, and SS)). Regarding moisture, CN milk was the only vegetable milk that exhibited moisture levels (91.3 g 100 g^−1^) like the blends (91.8–93.0 g 100 g^−1^). Additionally, the S milk and H presented the highest moisture content (91.6 and 95.0 g 100 g^−1^, respectively), exceeding that of the designed plant-based milk (91.3–93.0 g 100 g^−1^). Regarding ash content, S had the highest value (0.374 g 100 g^−1^), and its presence in the designed plant-based milk resulted in higher ash content (0.299 g 100 g^−1^), as can be seen in sample F. Despite containing 17.39% S, sample V exhibited a lower ash content (0.215 g 100 g^−1^), which may be attributed to the presence of 13.04% H, which had the lowest ash content among the ingredients. The designed plant-based milk (L) had a considerable ash content (0.336 g 100 g^−1^), which was one of the highest values obtained.

Regarding protein content, it was noted that both blends containing S and CN nuts achieved high values of this component (up to 1.3 g 100 g^−1^), which has already been verified in beverages produced using the ingredients separately. This outcome aligns with the conclusions of Wang, Cabral and Fernandes [18] and Fernandes et al. [62], who demonstrated that an increase in S content in the blend resulted in a higher protein content. The lower protein content in mixture L (0.7 g 100 g^−1^) could be attributed to the presence of SS in its composition, as it is the sample with the lowest protein content.

Regarding lipid content, sample L comprised 85.71% CN (3.6 g 100 g^−1^); thus, it exhibited a lipid content like that of individual CN milk. Conversely, sample F showed a lipid content similar to that of sample L and CN (3.4 and 3.6 g 100 g^−1^, respectively), despite containing less CN content. This higher lipid content was attributed to the presence of BNs in their composition, which naturally have a high lipid content. Furthermore, sample V had the lowest lipid content (2.7 g 100 g^−1^) among the designed plant-based milk, aligning with the lipid contents of S and HN present in its composition (2.6 and 2.7 g 100 g^−1^, respectively).

Concerning viscosity, the blend L had the highest viscosity of all the blends (27.3 cp), which was significantly different from all others. This viscosity seemed to be associated with the presence of CN, as they represented 85.7% of the formulation, and when used in isolation, it showed a similar viscosity (23.3 cp) related to its high dietary fiber content, that impacted viscosity [63]. The other two blends (V and F) presented lower viscosity values (10.8 and 9.4 cp, respectively) possibly due to the influence of the other components present in the mixtures, especially the lower concentration of CN and the greater presence of raw material with low influence on viscosity when used separately.

**Figure 5:**
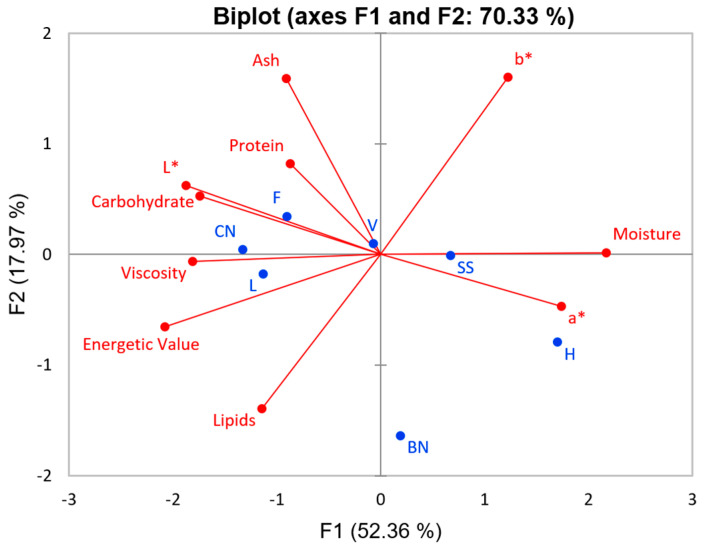
Principal components analysis of plant-based milk and designed plant-based milk. (PC1 x PC2). Active variables (●) and observations (●).

The principal component results are shown in Figure 5. The first two principal components explained 70.33% of the total variance. Here, the mixtures and CN milk were similar. Notably, some of the most important characteristics of the designed plant-based milk and CN milk are their viscosity, carbohydrate, L*, and protein. According to the percentages of CN in each milk sample (V = 69.57%, F = 57.14%, and L = 85.71%), the L sample was expected to be more similar to CN milk, which was noticeable by its proximity. Consequently, sample V was anticipated to exhibit closer proximity to CN milk than sample F. However, the presence of other components in sample V, namely, H and S, tended to influence the characteristics of the sample. Because these components are positioned on opposite sides of the graph, sample V appears to be more centralized.

Table 7 compares the averages of the analyzed variables of the groups formed in the cluster analysis, which can be observed in the main characteristics of each group. Group 1 was characterized by the highest viscosity values. Group 3 exhibited the lowest L* values. Group 4 exhibited the highest ash content and b* value. Moreover, it was still possible to observe that many variables did not vary significantly.

### 3.6. Dispersion Stability of Plant-Based Milk and Designed Plant-Based Milk

Figure 3 shows the dispersion stability of the plant-based milk and designed plant-based milk after 1 d of storage at 4 °C and 24 °C. Stability depends on the raw material, extraction method, formulation, and storage conditions because it is a colloidal suspension of dissolved and disintegrated plant-based materials [6,64].

In general, the use of raw material combinations seemed to improve dispersion stability compared with plant-based milk produced with a single ingredient. Raw materials influenced plant-based milk stability. The designed formulation F showed no difference in stability between both temperatures, but among the three designed formulations, plant-based milk had the lowest stability, separating by up to 77.5%. This separation could be associated with the lower concentration of CN present in the blend composition (38.10%). Additionally, blend L [85.71% CN, 9.52% SS, and 4.76% BN] exhibited complete stability at both temperatures. These findings were attributed to the presence of CN, as this ingredient was a key compound in the stability of plant-based milks, which was related to their high dietary fiber content [63].

Dispersion stability plays a crucial role in the quality of cow milk, as ensured by techniques such as homogenization and the use of additives, such as citrate [65]. The adoption of these procedures renders the product stable throughout the storage period and can be extended up to six months without phase separation. These practices can be adapted and implemented for plant-based milk to ensure comparable stability and preserve the integrity of the product.

### 3.7. Mineral Composition of Cow’s Milk and Designed Plant-Based Milk

The results of the mineral analysis comparing the designed plant-based milk with cow milk are presented in Table 8.

In general, cow milk was observed to have higher levels of minerals, except for the presence of Fe and Mg. However, based on the importance of intake and previously reported health impacts, the development of formulations should be continuously promoted as closely as possible to meet individual nutritional needs. The plant-based milk L [85.71% CN, 9.52% SS, and 4.76% BN] exhibited the highest Fe content (2.79 mg/L^−1^) due to the higher proportion of CN used, which had already shown a higher concentration of Fe when used alone (3.74 mg/L; Table 4). Except for whole milk C, cow milk had the highest Ca content (up to 1030.0 mg/L^−1^), a component naturally present at high concentrations in this type of product. In terms of K concentration, the plant-based milk F [57.14% CN, 38.10% BN, and 4.76% S] was statistically similar to the cow milk samples (1710.0 and 2390.0 mg/L^−1^, respectively), as it was formulated with ingredients naturally rich in K. Regarding Mg, sample F had higher levels (217.0 mg/L^−1^) than the cow milk samples (up to 81.9 mg/L^−1^), due to the use of large proportions of CN and BN, which are rich in Mg. For zinc, except for the whole milk A sample (8.17 mg/L^−1^), all other samples had statistically similar values (1.5–3.49 mg/L^−1^). For P, none of the developed samples were similar to the evaluated cow milk (100.0–367.7 and 428.0–668.0 mg/L^−1^, respectively).

Although the blends were designed to match the mineral concentration of cow milk, deficiencies were still observed, particularly in terms of calcium, potassium, and phosphorus. In general, the mineral concentration in plant-based milk could be adjusted by modifying the formulation or the types of vegetable sources and their proportions used in their preparation to achieve levels close to those found in the commonly used cow milk. Additionally, exogenous mineral supplementation may be considered to address deficiencies and standardize plant-based milk.

### 3.8. Mineral Composition of the Plant-Based Milk and Designed Plant-Based Milk

A comparison of minerals in plant-based milk and the designed plant-based milk is presented in Table 8 (means within the same column with different uppercase letters between designed plant-based milk (L, V, and F) and plant-based milk (H, CN, BN, S, and SS)). In general, despite having lower mineral concentrations than cow milk, the blends showed an increase in the concentration of specific minerals when compared to some plant-based milk produced with a single ingredient. Therefore, in some cases, the nutritional profile improved. However, the formulation of the blends diluted some minerals found in larger proportions in certain plant materials. However, this method can be used to customize the nutritional profiles of beverages for specific target consumers. The values obtained for mixtures L and F were similar to those of BN and S, which had the highest contents. For mixture L, this value may be due to the presence of BN, and F could contain both BN and S. The V mixture had contents comparable to those of CN, H, and SS.

As for Fe, the L mixture, which had a higher CN content, had similar values, and the V and F mixtures, which had lower CN content, had lower values, indicating that the other components of the mixture influenced the Fe content. Regarding K, the sample with the highest content was mixture F, which was higher than that of its raw materials. In contrast, the L and V mixtures had similar contents to those of the plant-based milk studied.

Regarding Mg, the CN and the mixtures L and F presented values that did not differ significantly (202.0, 175.0, and 217.0 mg L^−1^, respectively). For L, this was due to the high content of CN (87.51%), and most of these characteristics were preserved. However, mixture F had the lowest CN content (57.1%) and presented similar levels (93.3 mg L^−1^), whereas mixture V had lower levels of Mg (93.3 mg L^−1^), indicating that S and H milk had a great influence on the sample. In relation to P and Zn, these were verified using the same method as the mineral Mg; both the CN water-soluble extract and the mixtures L and F had the highest contents of Mg. The mixture V exhibited a lower Mg content; in the case of P, the concentration was equivalent to the other plant-based milk studied, and in the case of Zn, it was similar to the BN and SS water-soluble extract. In most cases, the F mixture had a higher mineral content than the original raw materials, showing that this mixture has great potential.

Fernandes, Wang, Cabral and Borges [62] noted that the increase in the content of soybean in their blends led to an increase in the content of certain minerals (P, Ca, K, Mg, Fe, Cu, and Zn). Because mixture F had 4.76% S in its composition, a formulation with S, CN, and BN at different concentrations can be studied, increasing the soybean content to verify whether there would continue to be an improvement in the content of these minerals.

Limited studies regarding the supplementation of plant-based milk with vitamins and minerals are present. Most of these studies have focused on analyzing the nutritional composition of commercially available plant-based products, as observed by Brooker et al. [66] who reported that approximately 65.0% of plant-based milk products sold in Australia and Singapore were fortified to address mineral deficiencies, with calcium being the most commonly used mineral. Similarly, Craig and Fresán [67] reported that more than half of the plant-based milk commercially available in the USA, Australia, and Western Europe is fortified, especially with Ca concentrations equal to or higher than those in cow milk. However, Zhang et al. [68] reported the production of oat milk with common hydrothermal treatments and fortification with Ca, P, Fe, Zn, and Se to improve its nutritional profile. The authors emphasized the importance of properly selecting and controlling the process to better retain minerals during the storage period.

### 3.9. Shelf Life Analysis

The results of viscosity, pH, and the microbiology profile were considered to evaluate the shelf life of designed plant-based milk (Tables S1–S3, respectively) at 0, 5, and 12 d in storage at 4 °C and 24 °C, respectively.

The mesophilic analysis only occurred until the sixth day, as the microbial growth was already greater than 10^6^ CFU/mL in the plates, and the samples presented a very strong odor and gas formation. This was expected because the products did not receive any additional conservation treatments. The deterioration of food by mesophiles occurs because of the growth of mesophilic microorganisms, such as bacteria and fungi, under moderate temperature conditions. This leads to sensory changes in the food and poses health risks if toxins are produced. Therefore, regardless of the product, proper storage, refrigeration, and hygiene practices are recommended to prevent this deterioration [69].

At 4 °C, the only plant-based milk that exhibited a growth of more than 10 CFU/mL was the H one, which showed a growth of 10^3^ on the sixth day. On day 11, no growth of more than 10 CFU/mL was observed in any of the plant-based milk products. In the case of H milk, microorganisms may have caused a decline and death of the microbial growth curve. Regarding the designed plant-based milk, as well as the other plant-based milk, no significant growth was observed until day 11, indicating that they were microbiologically stable at a temperature of 4 °C for 11 days. Machado [22] found that a pasteurized BN and macadamia beverage had a shelf life of 28 d under refrigeration, more than double that presented here; however, none had undergone any kind of heat treatment. However, this storage duration is also shorter than the average of six months achieved for commercially available cow milk subjected to ultra-high temperature processing (UHT) and up to 10 d for pasteurized milk.

A daily difference in viscosity between plant-based milk and their mixtures was observed. No pattern of viscosity change was observed, neither an increase nor a decrease. CN milk showed a decrease in viscosity throughout the analysis under both storage conditions, whereas the other plant-based beverages showed stable conditions or an increase in viscosity. However, the plant-based milk exhibited no changes in viscosity. Some bacteria are capable of hydrolyzing polysaccharides, while others are capable of synthesizing them, resulting in variation in viscosity, in the first case leading to a decrease and, in the second, an increase. This suggests that the microbiota present in the CN beverage is different from that present in other beverages and that the microbiota of a mixture of plant-based water-soluble extracts varies with storage conditions [69].

With regard to the pH, a pattern of alteration was observed during storage. An increase in the parameter was noted on the fifth and twelfth day of storage at 4 °C, especially for the designed plant-based milk (L and V blends). For samples stored at 24 °C, a decrease in pH was observed in all cases, which was related to medium acidification owing to microbial growth [70]. Based on what was presented, evaluating plant-based milk production is essential to ensure the better quality of the final product, the standardization of the manufacturing process, improvement in operational efficiency, meeting regulatory requirements, satisfying consumers, and promoting sustainability.

The limitations of this study are the absence of preservatives and stricter conservation methods, which are essential for prolonging the shelf life of products. Future research should focus on the development and application of natural preservatives and innovative conservation techniques to improve durability, without compromising product safety or quality. Furthermore, achieving a balanced nutritional profile from ingredient mixtures is challenging because it requires a careful balance among the various nutritional components, making the process complex and subject to many variables.

## 4. Conclusions

The utilization of various raw materials facilitated the development of plant-based milks with diverse characteristics and compositions. However, these alternatives did not provide the complete nutritional profile offered by cow’s milk. Plant-based milk produced from isolated grains exhibited a generally inferior nutritional composition compared to cow’s milk, particularly in protein content (up to 1.1 g/100 g), lipids (up to 2.7 g/100 g), color parameters, and minerals, especially calcium (up to 62.4 mg/L compared to cow’s milk’s 1030 mg/L). Conversely, plant-based milk formulated using a blend of ingredients demonstrated improved nutritional profiles, with enhancements in minerals, high-quality lipids (up to 3.6 g/100 g), and carbohydrates (3.4 g/100 g using CN, BN, and S). While the protein content remained lower at 1.3% compared to 5.7% in cow’s milk, the caloric value of plant-based milk (32.8 to 52.1 kcal) was similar to that of cow’s milk. Additionally, satisfactory microbiological stability was observed over an 11-day storage period at 4 °C. Future research should focus on developing natural preservatives and innovative conservation techniques to improve durability without compromising safety and quality. Balancing the nutritional profile of mixed ingredients remains complex due to the need for precise nutritional balance.

## Figures and Tables

**Table 1:** Composition of designed plant-based milk produced from a blend of hazelnut, Brazil nut, cashew nut, soybean, and sunflower seed.

Blend	Brazil Nut (BN)	Cashew Nut (CN)	Hazelnut (H)	Soy (S)	Sunflower Seed (SS)
L (BN + CN + SS)	4.76%	85.71%	-	-	9.52%
V (CN + H + S)	-	69.57%	13.04%	17.39%	-
F (BN + CN + S)	38.10%	57.14%	-	4.76%	-

**Table 2:** Moisture, ash, protein, lipids, carbohydrate, energetic value, viscosity, and color parameters.

Sample	Moisture	Ash	Protein	Lipids	Carbohydrate	Energetic Value	Viscosity	Color		
	(g 100 g^−1^)					(kcal)	(cp)	L*	a*	b*
Hazelnut	95.0 ^a^ ± 0.5	0.117 ^d^ ± 0.03	0.744 ^e^ ± 0.1	2.7 ^c^ ± 0.10	1.71 ± 0.1	32.8 ± 2.00	4.30 ^f^ ± 0.04	62.0 ^f^ ± 1.72	4.86 ^a^ ± 0.80	10.9 ^b^ ± 1.66
Cashew nut	91.25 ^c,d^ ± 0.0	0.235 ^c,d^ ± 0.02	1.17 ^e^ ± 0.01	3.6 ^b^ ± 0.10	3.74 ± 0.1	52.1 ± 0.597	23.3 ^a^ ± 0.2	73.0 ^d^ ± 0.01	−0.547 ^c^ ± 0.015	8.723 ^c^ ± 0.1
Brazil nut	93.9 ^b^ ± 0.1	0.213 ^d^ ± 0.0	0.875 ^e^ ± 0.12	4.5 ^a^ ± 0.05	0.443 ± 0.3	46.1 ± 0.197	3.94 ^g^ ± 0.02	68.7 ^e^ ± 0.19	1.14 ^b^ ± 0.19	6.64 ^d^ ± 0.42
Soy	94.6 ^a,b^ ± 0.2	0.374 ^c^ ± 0.03	1.13 ^e^ ± 0.20	2.6 ^c^ ± 0.23	1.58 ± 0.5	32.8 ± 0.659	4.70 ^b,c,d^ ± 0.1	69.0 ^e^ ± 0.88	1.39 ^b^ ± 0.17	15.7 ^a^ ± 0.18
Sunflower seed	94.8 ^a^ ± 0.1	0.250 ^c,d^ ± 0.04	0.392 ^e^ ± 0.1	2.8 ^c^ ± 0.12	1.77 ± 0.1	33.5 ± 1.14	4.48 ^e,f^ ± 0.1	68.9 ^e^ ± 0.11	−0.470 ^c^ ± 0.14	8.46 ^c^ ± 0.43
Whole milk A	88.3 ^e,f^ ± 0.2	0.616 ^b^ ± 0.03	4.37 ^c^ ± 0.33	3.3 ^b^ ± 0.29	3.34 ± 0.1	60.8 ± 2.11	4.80 ^b,c^ ± 0.1	82.6 ^a^ ± 0.1	−2.86 ^d^ ± 0.02	7.67 ^c,d^ ± 0.01
Skimmed milk A	91.2 ^c,d^ ± 0.1	0.623 ^b^ ± 0.04	5.50 ^a,b^ ± 0.2	0.00 ^e^ ± 0.00	2.66 ± 0.3	32.6 ± 0.198	4.61 ^c,d,e^ ± 0.1	75.0 ^c^ ± 0.1	−4.76 ^e^ ± 0.03	2.70 ^e^ ± 0.1
Whole milk B	88.9 ^e^ ± 0.55	0.768 ^a,b^ ± 0.01	2.75 ^d^ ± 0.0	3.3 ^b^ ± 0.30	4.27 ± 0.3	57.8 ± 3.53	4.89 ^b^ ± 0.02	81.9 ^a^ ± 0.01	−3.11 ^d^ ± 0.01	7.54 ^c,d^ ± 0.1
Skimmed milk B	91.7 ^c^ ± 0.02	0.793 ^a^ ± 0.02	5.75 ^a^ ± 0.54	0.00 ^e^ ± 0.00	2.05 ± 0.03	30.0 ± 0.167	4.63 ^c,d,e^ ± 0.02	74.2 ^c,d^ ± 0.01	−5.16 ^e^ ± 0.0	2.12 ^e^ ± 0.01
Whole milk C	88.1 ^f^ ± 0.1	0.675 ^a,b^ ± 0.2	6.25 ^a^ ± 0.45	3.3 ^b^ ± 0.10	1.85 ± 0.16	62.1 ± 1.23	4.91 ^b^ ± 0.04	82.8 ^a^ ± 0.04	−2.56 ^d^ ± 0.02	7.55 ^c,d^ ± 0.02
Skimmed milk C	90.6 ^d^ ± 0.05	0.819 ^a^ ± 0.01	4.81 ^b,c^ ± 0.44	0.47 ^d^ ± 0.1	3.26 ± 0.42	36.7 ± 0.148	4.55 ^d,e^ ± 0.03	78.0 ^b^ ± 0.01	−3.19 ^d^ ± 0.01	6.85 ^d^ ± 0.01

^a–g^ Means within the same column with different letters are significantly different (Tukey’s test, *p* < 0.05).

**Table 3:** Moisture, ash, protein, lipids, carbohydrate, energetic value, viscosity, and color parameters of the groups formed.

Group	Moisture	Ash	Protein	Lipids	Carbohydrate	Energetic Value	Viscosity	Color		
	(g 100 g^−1^)					(kcal)	(cp)	L*	a*	b*
1	89.1 ^c^ ± 1.33	0.584 ^a^ ± 0.229	3.63 ^b^ ± 1.98	3.38 ^a^ ± 0.229	3.30 ± 0.953	58.2 ± 4.45	9.48 ^a^ ± 8.35	80.0 ^a^ ± 4.29	−2.27 ^b^ ± 1.06	7.87 ^b^ ± 0.517
2	91.2 ^b^ ± 0.486	0.738 ^a^ ± 0.09	5.42 ^a^ ± 0.532	0.156 ^b^ ± 0.235	2.66 ± 0.545	33.1 ± 2.92	4.60 ^a^ ± 0.04	75.8 ^b^ ± 1.74	−4.37 ^c^ ± 0.900	3.89 ^c^ ± 2.23
3	94.6 ^a^ ± 0.513	0.250 ^b^ ± 0.09	0.786 ^c^ ± 0.300	3.14 ^a^ ± 0.851	1.46 ± 0.524	36.3 ± 6.00	4.36 ^a^ ± 0.29	67.3 ^c^ ± 3.32	1.73 ^a^ ± 2.06	10.4 ^a^ ± 3.61

^a–c^ Means within the same column with different letters are significantly different (Tukey’s test, *p* < 0.05). (Group 1) whole milk and cashew beverage; (Group 2) skim milk; (Group 3) hazelnuts, Brazil nuts, sunflower seeds, and soy.

**Table 4:** Mineral composition of cow’s milk and plant-based milk. Means within the same column with different letters are significantly different (Tukey test *p* < 0.05).

Sample	Ca	Fe	K	Mg	P	Zn
	(mg L^−1^)					
Hazelnut	4.14 ^d^ ± 1.93	1.13 ^bc^ ± 0.483	<25.2 ^d^	36.2 ^c^ ± 9.27	26.7 ^f^ ± 3.97	0.883 ^c^ ± 0.09
Cashew nut	12.3 ^d^ ± 3.72	3.74 ^a^ ± 0.05	424.0 ^cd^ ± 18.2	202.0 ^a^ ± 2.77	342.0 ^e^ ± 10.1	3.79 ^b^ ± 0.02
Brazil nut	62.4 ^d^ ± 19.3	1.85 ^b^ ± 1.02	315.0 ^d^ ± 22.5	141.0 ^b^ ± 40.2	129.0 ^f^ ± 56.9	1.72 ^bc^ ± 0.252
Soy	62.3 ^d^ ± 21.5	0.636 ^cd^ ± 0.01	466.0 ^cd^ ± 25.2	63.7 ^c^ ± 7.85	112.0 ^f^ ± 5.65	1.24 ^c^ ± 0.563
Sunflower seed	12.4 ^d^ ± 1.71	1.21 ^bc^ ± 0.06	862.0 ^cd^ ± 61.0	84.4 ^c^ ± 17.5	79.5 ^f^ ± 54.2	1.37 ^c^ ± 0.189
Whole milk A	1030 ^a^ ± 138	<5.40 ^d^ × 10^−3^	1190 ^bc^ ± 141.0	78.1 ^c^ ± 24.4	668.0 ^ab^ ± 87.7	8.17 ^a^ ± 2.09
Skimmed milk A	902 ^ab^ ± 31.0	<5.40 ^d^ × 10^−3^	1370.0 ^bc^ ± 46.5	81.9 ^c^ ± 2.95	616.0 ^bc^ ± 36.6	1.76 ^c^ ± 0.08
Whole milk B	930 ^ab^ ± 79.6	<5.40 ^d^ × 10^−3^	2390.0 ^a^ ± 519.0	73.4 ^c^ ± 13.3	778.0 ^a^ ± 64.3	2.29 ^bc^ ± 0.177
Skimmed milk B	768 ^b^ ± 63.0	<5.40 ^d^ × 10^−3^	1440 ^b^ ± 68.0	66.3 ^c^ ± 2.25	596.0 ^bcd^ ± 48.9	2.28 ^bc^ ± 0.378
Whole milk C	300 ^c^ ± 19.3	<2.89 ^d^ × 10^−2^	1070.0 ^bc^ ± 260.0	46.2 ^c^ ± 27.8	428.0 ^de^ ± 30.5	1.59 ^c^ ± 0.758
Skimmed milk C	820 ^b^ ± 81.5	<5.40 ^d^ × 10^−3^	1400 ^b^ ± 17.7	68.0 ^c^ ± 2.22	506.0 ^bc^ ± 59.2	2.42 b^c^ ± 0.295

**Table 5:** Moisture, ash, protein, lipids, carbohydrate, energetic value, viscosity, and color parameters of the designed plant-based milk and cow’s milk.

Sample	Moisture	Ash	Protein	Lipids	Carbohydrate	Energetic Value	Viscosity	Color		
	(g 100 g^−1^)					(kcal)	(cp)	L*	a*	b*
L	91.8 ^b,cD^ ± 0.07	0.346 ^cAB^ ± 0.05	0.777 ^eC^ ± 0.07	3.6 ^aB^ ± 0.15	3.40 ± 0.278	49.8 ± 0.7	27.3 ^aA^ ± 0.0	70.1 ^gB^ ± 0.01	−0.08 ^bCD^ ± 0.01	6.76 ^bE^ ± 0.04
V	93.0 ^aC^ ± 0.2	0.215 ^cCD^ ± 0.115	1.30 ^eA^ ± 0.07	2.7 ^bC^ ± 0.1	2.68 ± 0.146	41.2 ± 2.2	10.8 ^bC^ ± 0.1	67.9 ^hC^ ± 0.25	0.510 ^aBC^ ± 0.1	6.97 ^bDE^ ± 0.410
F	91.9 ^bC^ ± 0.1	0.299 ^cA,B,C^ ± 0.01	1.14 ^eAB^ ± 0.07	3.4 ^aB^ ± 0.17	3.19 ± 0.293	48.3 ± 1.1	9.45 ^cD^ ± 0.1	73.5 ^fA^ ± 0.21	−0.230 ^cCD^ ± 0.1	7.61 ^aDE^ ± 0.320
Whole milk A	88.3 ^e,f^ ± 0.2	0.616 ^b^ ± 0.03	4.37 ^c^ ± 0.33	3.3 ^a^ ± 0.29	3.34 ± 0.1	60.8 ± 2.1	4.80 ^d^ ± 0.05	82.6 ^a^ ± 0.1	−2.86 ^e^ ± 0.2	7.67 ^a^ ± 0.01
Skimmed milk A	91.2 ^c,d^ ± 0.1	0.623 ^b^ ± 0.04	5.50 ^a,b^ ± 0.23	0.00 ^c^ ± 0.00	2.66 ± 0.28	32.6 ± 0.1	4.61 ^e^ ± 0.05	75.0 ^d^ ± 0.10	−4.76 ^g^ ± 0.03	2.70 ^c^ ± 0.1
Whole milk B	88.9 ^e^ ± 0.5	0.768 ^a,b^ ± 0.01	2.75 ^d^ ± 0.00	3.3 ^a^ ± 0.30	4.27 ± 0.33	57.8 ± 3.5	4.89 ^d^ ± 0.01	81.9 ^b^ ± 0.01	−3.11 ^f^ ±0.02	7.54 ^a^ ± 0.1
Skimmed milk B	91.7 ^b,c^ ± 0.02	0.793 ^a,b^ ± 0.02	5.75 ^a,b^ ± 0.54	0.00 ^c^ ± 0.00	2.05 ± 0.03	30.0 ± 0.1	4.63 ^e^ ± 0.02	74.2 ^e^ ± 0.01	−5.16 ^h^ ± 0.0	2.12 ^d^ ± 0.01
Whole milk C	88.1 ^f^± 0.1	0.675 ^a,b^ ± 0.198	6.25 ^a^ ± 0.45	3.3 ^a^ ± 0.10	1.85 ± 0.163	62.1 ± 1.23	4.91 ^d^ ± 0.04	82.8 ^a^ ± 0.04	−2.56 ^d^ ± 0.02	7.55 ^a^ ± 0.02
Skimmed milk C	90.6 ^d^ ± 0.05	0.819 ^a^ ± 0.01	4.81 ^b,c^ ± 0.44	0.47 ^c^ ± 0.05	3.26 ± 0.423	36.7 ±0.148	4.55 ^e^ ± 0.04	78.0 ^c^ ± 0.01	−3.19 ^f^ ± 0.01	6.85 ^b^ ± 0.01
Hazelnut (H)	95.0 ^A^ ±0.6	0.117 ^D^ ± 0.03	0.744 ^C^ ± 0.08	2.7 ^C^ ± 0.10	1.71 ± 0.08	32.8 ± 2.00	4.30 ^F^ ± 0.04	62.0 ^D^ ± 1.7	4.86 ^A^ ± 0.800	10.9 ^B^ ± 1.66
Cashew nut (CN)	91.3 ^D^ ± 0.0	0.235 ^B,C,D^ ± 0.02	1.17 ^A,B^ ± 0.01	3.6 ^B^ ± 0.10	3.74 ± 0.07	52.1 ± 0.59	23.3 ^B^ ± 0.208	73.0 ^A^ ± 0.01	−0.547 ^D^ ± 0.01	8.72 ^C,D^ ± 0.06
Brazil nut (BN)	93.9 ^B^ ± 0.1	0.213 ^C,D^ ± 0.01	0.875 ^B,C^ ± 0.12	4.5 ^A^ ± 0.05	0.611 ± 0.06	46.1 ± 0.19	3.94 ^G^ ± 0.02	68.7 ^B,C^ ± 0.2	1.14 ^B^ ± 0.196	6.64 ^E^ ± 0.422
Soy (S)	94.6 ^A^ ± 0.2	0.374 ^A^ ± 0.03	1.13 ^A,B^ ± 0.20	2.6 ^C^ ± 0.23	1.58 ± 0.53	32.8 ± 0.65	4.70 ^E^ ± 0.06	69.0 ^B,C^ ± 0.9	1.39 ^B^ ± 0.172	15.7 ^A^ ± 0.181
Sunflower seed (SS)	94.8 ^A^ ± 0.13	0.250 ^B,C,D^ ± 0.04	0.392 ^D^ ± 0.05	2.8 ^C^ ± 0.12	1.77 ± 0.11	33.5 ± 1.14	4.48 ^E,F^ ± 0.05	68.9 ^B,C^ ± 0.1	−0.470 ^D^ ± 0.139	9.52 ^B,C^ ± 0.501

Means within the same column with different lowercase letters are significantly different (Tukey’s test, *p* < 0.05) between designed plant-based milk (L: 85.71% CN + 9.52% SS + 4.76% BN; V: 69.57% CN + 17.29% S + 13.04% H; F: 57.14% CN + 38.10% BN + 4.76% S) and different cow’s milk; means within the same column with different uppercase letters are significantly different (Tukey’s test, *p* < 0.05) between designed plant-based milk (L, V and F) and plant-based milk (H, CN, BN, S, and SS).

**Table 6:** Moisture, ash, protein, lipids, carbohydrate, energetic value, viscosity, and color of the groups formed.

Group	Moisture	Ash	Protein	Lipids	Carbohydrate	Energetic Value	Viscosity	Color		
		(g 100 g^−1^)				(kcal)	Viscosity (cp)	L*	a*	b*
1	92.2 ^a^ ± 0.588	0.283 ^b^ ± 0.07	1.07 ^b^ ± 0.247	3.2 ^a^ ± 0.445	3.09 ± 0.385	46.3 ± 4.29	15.9 ^a^ ± 8.60	70.5 ^c^ ± 2.43	0.130 ^a^ ± 0.365	7.11 ^a^ ± 0.464
2	91.2 ^b^ ± 0.486	0.738 ^a^ ± 0.09	5.42 ^a^ ± 0.532	0.16 ^b^ ± 0.235	2.66 ± 0.545	33.1 ± 2.92	4.60 ^b^ ± 0.04	75.8 ^b^ ± 1.74	−4.37 ^c^ ± 0.900	3.89 ^b^ ± 2.23
3	88.4 ^c^ ± 0.479	0.700 ^a^ ± 0.105	4.46 ^a^ ± 1.54	3.3 ^a^ ± 0.215	3.15 ± 1.07	60.2 ± 2.89	4.87 ^b^ ± 0.06	82.4 ^a^ ± 0.395	−2.84 ^b^ ± 0.238	7.59 ^a^ ± 0.06

^a–c^ Means within the same column with different letters are significantly different (Tukey’s test, *p* < 0.05). (Group 1) designed plant-based milk [L 85.71% CN, 9.52% SS, 4.76% BN] [V 69.57% CN, 17.29% S, 13.04% H] [F 57.14% CN, 38.10% BN, 4.76% S], (Group 2) whole milk, (Group 3) skimmed milk.

**Table 7:** Mean values of moisture, ash, protein, lipids, carbohydrate, energetic value, and viscosity of the groups formed.

Group	Moisture	Ash	Protein	Lipids	Carbohydrate	Energetic Value	Viscosity
	(g 100 g^−1^)					(kcal)	(cp)
1	92.0 ^b^ ± 0.6	0.271 ^b^ ± 0.06	1.11 ^a^ ± 0.20	3.3 ^a^ ± 0.4	3.25 ± 0.44	47.7 ± 4.49	17.7 ^a^± 8.08
2	94.4 ^a^ ± 0.5	0.231 ^bc^ ± 0.03	0.633 ^b^ ± 0.27	3.7 ^a^ ± 0.9	1.31 ± 0.64	40.8 ± 7.18	4.21 ^b^ ± 0.296
3	95.0 ^a^ ± 0.6	0.117 ^c^ ± 0.03	0.744 ^ab^ ± 0.08	2.7 ^a^ ± 0.1	1.71 ± 0.08	31.8 ± 1.42	4.30 ^b^ ± 0.04
4	94.6 ^a^ ± 0.2	0.374 ^a^ ± 0.03	1.13 ^a^ ± 0.20	2.6 ^a^ ± 0.2	1.58 ± 0.52	33.0 ± 0.737	4.70 ^b^ ± 0.06

^a–c^ Means within the same column with different letters are significantly different (Tukey’s test, *p* < 0.05). (Group 1: mixture of plant-based milk [L 85.71% CN, 9.52% SS, 4.76% BN] [V 69.57% CN, 17.29% S, 13.04% H] [F 57.14% CN, 38.10% BN, 4.76% S]; Group 2: Brazil nut and sunflower seed milk; Group 3: hazelnut milk; Group 4: soy milk.

**Table 8:** Mineral composition (mg L^−1^) of cow’s milk and designed plant-based milk.

Sample	Ca	Fe	K	Mg	P	Zn
L	27.8 ^bcABC^ ± 12.4	2.79 ^aAB^ ± 0.3	351.0 ^cBC^ ± 20.3	175.0 ^aAB^ ± 2.31	289.0 ^cdA^ ± 2.02	3.49 ^bA^ ± 0.20
V	10.4 ^cC^ ± 0.14	2.02 ^bBC^ ± 0.2	340.0 ^cBC^ ± 66.5	93.3 ^bCD^ ± 1.09	100.0 ^dB^ ± 7.38	2.18 ^bB^ ± 0.2
F	52.1 ^bcAB^ ± 1.14	2.25 ^bBC^ ± 0.1	1710.0 ^abA^ ± 264.0	217.0^2 aA^ ± 10.3	367.0 ^bcdA^ ± 30.3	3.84 ^bA^ ± 0.5
Whole milk A	1030.0 ^a^ ± 138.0	<5.40 ^c^ × 10^−3^	1190.0 ^b^ ± 141.0	78.1 ^b^ ± 24.4	668.0 ^ab^ ± 87.7	8.17 ^a^ ± 2.0
Skim milk A	902.0 ^a^ ± 31.0	<5.40 ^c^ × 10^−3^	1370.0 ^b^ ± 46.5	81.9 ^b^ ± 2.95	616.0 ^ab^ ± 36.6	1.76 ^b^ ± 0.1
Whole milk B	930.0 ^a^ ± 79.6	<5.40 ^c^ × 10^−3^	2390.0 ^a^ ± 519.0	73.4 ^b^ ± 13.3	778.0 ^a^ ± 64.3	2.29 ^b^ ± 0.18
Skim milk B	768.0 ^a^ ± 63.0	<5.40 ^c^ × 10^−3^	1140.0 ^b^ ± 68.0	66.3 ^b^ ± 2.25	596.0 ^abc^ ± 48.9	2.28 ^b^ ± 0.37
Whole milk C	300.0 ^b^ ± 19.3	<2.89 ^c^ × 10^−2^	1070.0 ^bc^ ± 260	46.2 ^b^ ± 27.8	428.0 ^abc^ ± 30.0	1.59 ^b^ ± 0.76
Skim milk C	820 ^a^ ± 81.5	<5.40 ^c^ × 10^−3^	1400.0^3 b^ ± 17.7	68.0 ^b^ ± 2.22	506.0 ^abc^ ± 59.2	2.42 ^b^ ± 0.3
Hazelnut (H)	4.14 ^C^ ± 1.93	1.13 ^CD^ ± 0.5	<25.2 ^C^	36.2 ^D^ ± 9.27	26.7 ^B^ ± 3.97	0.883 ^C^ ± 0.1
Cashew nut (CN)	12.3 ^BC^ ± 3.72	3.74 ^A^ ± 0.05	424.0 ^BC^ ± 18.2	202.0 ^A^ ± 2.77	342.0 ^A^ ± 10.1	3.79 ^A^ ± 0.02
Brazil nut (BN)	62.4 ^A^ ± 19.3	1.85 ^BC^ ± 1.02	315.0 ^BC^ ± 22.5	141.0 ^BC^ ± 40.2	129.0 ^B^ ± 56.9	1.72 ^BC^ ± 0.25
Soy (S)	62.3 ^A^ ± 21.5	0.636 ^D^ ± 0.01	466.02 ^BC^ ± 25.2	63.7 ^D^ ± 7.85	112.02 ^B^ ± 5.65	1.24 ^C^ ± 0.56
Sunflower Seed (SS)	12.4 ^BC^ ± 1.71	1.21 ^CD^ ± 0.1	862.0 ^B^ ± 61.0	84.4 ^D^ ± 17.5	79.5 ^B^ ± 54.2	1.37 ^BC^ ± 0.19

Means within the same column with different lowercases letters are significantly different (Tukey’s test, *p* < 0.05) between designed plant-based milk (L: 85.71% CN + 9.52% SS + 4.76% BN; V: 69.57% CN + 17.29% S + 13.04% H; F: 57.14% CN + 38.10% BN + 4.76% S) and different cow’s milk; means within the same column with different uppercase letters are significantly different (Tukey’s test, *p* < 0.05) between designed plant-based milk (L, V and F) and plant-based milk (H, CN, BN, S, and SS).

## Data Availability

The original contributions presented in the study are included in the article/Supplementary Materials, further inquiries can be directed to the corresponding author.

## Supplementary Materials

The following supporting information can be downloaded at: https://www.mdpi.com/article/10.3390/foods13142169/s1, Figure S1: Dendrograms of samples of plant-based milk substitutes (hazelnut (H), Brazil nut (BN), cashew nut (CN), sunflower seed (SS), and soy (S)), whole milk (N, EW, and QW), and skimmed milk (M, ES, and QS) obtained through the variables moisture, ash, proteins, lipids, carbohydrates, energetic value, viscosity, and color parameters; Figure S2: Dendrograms of samples of designed plant-based milk, whole milk, and skim milk obtained using the following variables: moisture, ash, proteins, lipids, carbohydrate, energetic value viscosity, and color parameters; Figure S3: Dendrograms of plant-based milk and designed plant-based milk obtained using variable moisture, ash, proteins, lipids, carbohydrates, energetic value, viscosity, and color parameters; Table S1: Mean viscosity values of samples during shelf life study; Table S2: Mean pH values of samples during the shelf life study; Table S3: Mean microbial count values of samples during the shelf life study.
