# Supplementary material for: A Comparative Study of Dairy and Non-Dairy Milk Types: Development and Characterization of Customized Plant-Based Milk Options
Source: Foods. 2024 Jul 9;13(14):2169. doi: 10.3390/foods13142169 (PMC11276104; doi:10.3390/foods13142169)
Supplement: Supplementary file 1 [file foods-13-02169-s001.zip › foods-3039860-supplementary.pdf]

A Comparative Study of Dairy and Non-Dairy Milk Types: Development and Characterization of Customized Plant-Based Milk Options

Aline Rolim Alves da Silva [1], Ricardo Erthal Santelli [1], Bernardo Ferreira Braz [1], Marselle Marmo Nascimento Silva [1], Lauro Melo [2], Ailton Cesar Lemes [2], Bernardo Dias Ribeiro [1,2,*]

[1] Instituto de Química, Universidade Federal do Rio de Janeiro, Av. Athos da Silveira Ramos, 149, Bloco A—Cidade Universitária, Rio de Janeiro 21044-020, RJ, Brazil; alinerolimas@gmail.com (A.R.A.d.S.); santelli@iq.ufrj.br (R.E.S.); bernardobraz@pos.iq.ufrj.br (B.F.B.); marsellemarmo@hotmail.com (M.M.N.S.)

[2] Escola de Química, Universidade Federal do Rio de Janeiro. Av. Athos da Silveira Ramos, 149, Bloco E—Cidade Universitária, Rio de Janeiro 21044-020, RJ, Brazil; lauro@eq.ufrj.br (L.M.); ailtonlemes@eq.ufrj.br (A.C.L.)

* Correspondence: bernardo@eq.ufrj.br

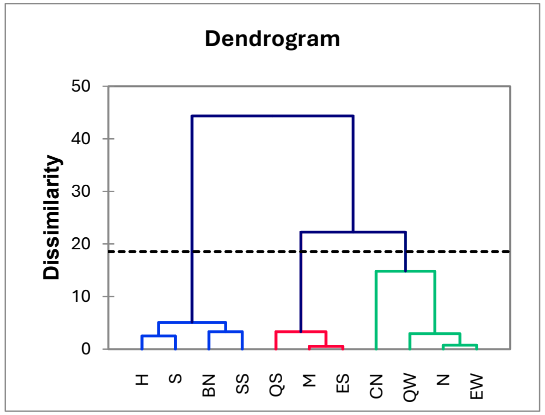

Figure S1. Dendrograms of samples of plant-based milk substitutes (hazelnut (H), Brazil nut (BN), cashew nut (CN), sunflower seed (SS), and soy (S)), whole milk (N, EW, and QW), and skimmed milk (M, ES, and QS) obtained through the variables moisture, ash, proteins, lipids, carbohydrates, energetic value, viscosity, and color parameters.

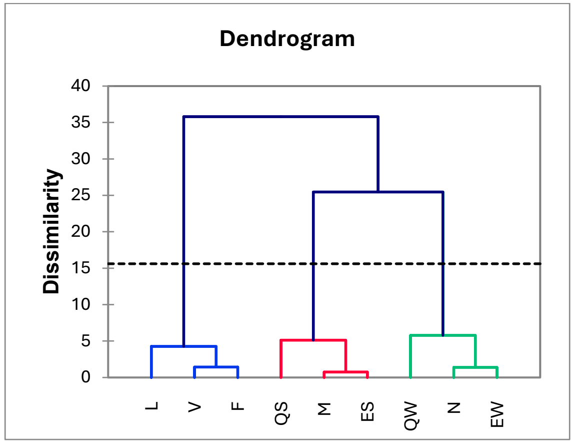

Figure S2. Dendrograms of samples of designed plant-based milk, whole milk, and skim milk obtained using the following variables: moisture, ash, proteins, lipids, carbohydrate, energetic value viscosity, and color parameters.

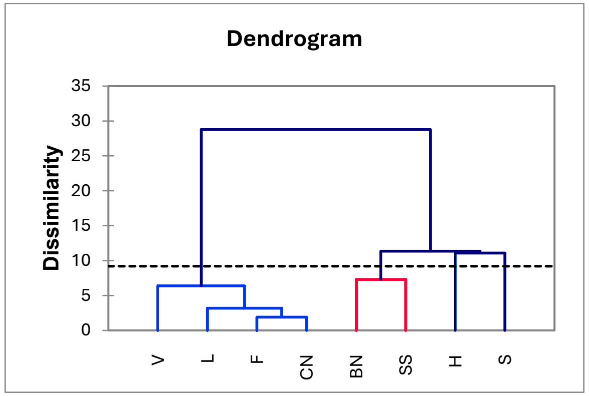

Figure S3. Dendrograms of plant-based milk and designed plant-based milk obtained using variable moisture, ash, proteins, lipids, carbohydrates, energetic value, viscosity, and color parameters.

Table S1. Mean viscosity values of samples during shelf life study.

	Day 0 (cp)	Day 5 (cp)		Day 11 (cp)
		4 ºC	24 ºC	4 ºC
L	$25.5^{B,b}\pm0.1$	$16.5^{B,d}\pm0.1$	$17.5^{B,c}\pm0.0$	$34.1^{A,a}\pm0.1$
V	$7.89^{D,c}\pm0.1$	$8.24^{C,b}\pm0.02$	$16.3^{C,a}\pm0.1$	$6.95^{D,d}\pm0.02$
F	$8.28^{C,b}\pm0.02$	$7.75^{D,c}\pm0.01$	$8.85^{D,a}\pm0.03$	$7.25^{C,d}\pm0.01$
S	$3.24^{F,c}\pm0.1$	$3.89^{E,b}\pm0.03$	$4.69^{F,a}\pm0.16$	$3.83^{E,b}\pm0.04$
CN	$50.8^{A,a}\pm0.3$	$31.6^{A,b}\pm0.3$	$26.7^{A,c}\pm0.15$	$23.4^{B,d}\pm0.1$
BN	$3.18^{F,b}\pm0.02$	$3.02^{F,c}\pm0.1$	$3.74^{G,a}\pm0.01$	$3.12^{G,b,c}\pm0.1$
H	$3.13^{F,d}\pm0.03$	$3.65^{E,c}\pm0.02$	$5.36^{E,a}\pm0.04$	$3.92^{E,b}\pm0.04$
SS	$3.91^{E,b}\pm0.1$	$3.70^{E,c}\pm0.1$	$4.69^{F,a}\pm0.03$	$3.62^{F,c}\pm0.0$

a-d Means within the same line with different letters are significantly different (Tukey test $p<0.05$)

A-G Means within the same column with different letters are significantly different (Tukey test $p<0.05$)

(H) Hazelnut; (CN) Cashew nut; (BN) Brazil nut; (S) Soy; (SS) Sunflower seed; (L) 85.71% CN, 9.52% SS, 4.76% BN; (V) 69.57% CN, 17.29% S, 13.04% H; (F) 57.14% CN, 38.10% BN, 4.76% S.

Table S2. Mean pH values of samples during shelf life study

	Day 0	Day 5		Day 11
		4 ºC	24 ºC	4 ºC
L	$6.63^{E,b}$±0.01	$6.71^{D,a}$±0.1	$5.00^{D,d}$±0.0	$6.29^{F,c}$± 0.01
V	$6.63^{E,c}$±0.05	$6.68^{E,a}$±0.0	$4.49^{D,E,d}$±0.0	$6.66^{D,b}$±0.0
F	$6.67^{D,b}$±0.0	$6.71^{D,a}$±0.01	$4.98^{E,c}$±0.0	$6.71^{B,a}$±0.0
S	$6.63^{E,a}$±0.05	$6.64^{F,a}$±0.01	$5.38^{A,c}$±0.01	$6.60^{E,b}$±0.01
CN	$6.62^{E,b}$±0.01	$6.66^{E,a}$±0.01	$4.72^{F,d}$±0.1	$6.60^{E,c}$±0.01
BN	$6.92^{B,a}$±0.01	$6.94^{B,a}$±0.01	$5.35^{B,c}$±0.1	$6.87^{A,b}$±0.0
H	$6.82^{C,b}$±0.01	$6.85^{C,a}$±0.0	$4.70^{F,d}$±0.01	$6.03^{G,c}$±0.0
SS	$6.99^{A,a}$±0.01	$6.98^{A,a}$±0.01	$5.27^{C,c}$±0.01	$6.68^{C,b}$±0.01

a-d Means within the same line with different letters are significantly different (Tukey test $p<0.05$). A-G Means within the same column with different letters are significantly different (Tukey test $p<0.05$). (H) Hazelnut; (CN) Cashew nut; (BN) Brazil nut; (S) Soy; (SS) Sunflower seed; (L) 85.71% CN, 9.52% SS, 4.76% BN; (V) 69.57% CN, 17.29% S, 13.04% H; (F) 57.14% CN, 38.10% BN, 4.76% S.

Table S3. Mean microbial count values of samples during shelf life study

	mesophilic		psychotropic		
	Day 0 (CFU/mL)	Day 5 (CFU/mL)	Day 0 (CFU/mL)	Day 5 (CFU/mL)	Day 11 (CFU/mL)
L	<10	$1{,}0X10^{6}$	<10	<10	<10
V	<10	$1{,}0X10^{6}$	<10	<10	<10
F	<10	$1{,}0X10^{6}$	<10	<10	<10
S	<10	$1{,}0X10^{6}$	<10	<10	<10
CN	<10	$1{,}0X10^{6}$	<10	<10	<10
BN	<10	$1{,}0X10^{6}$	<10	<10	<10
H	<10	$1{,}0X10^{6}$	<10	$2{,}3x10^{3}$	<10
SS	<10	$1{,}0X10^{6}$	<10	<10	<10

(H) Hazelnut; (CN) Cashew nut; (BN) Brazil nut; (S) Soy; (SS) Sunflower seed; (L) 85.71% CN, 9.52% SS, 4.76% BN; (V) 69.57% CN, 17.29% S, 13.04% H; (F) 57.14% CN, 38.10% BN, 4.76% S.
